# Barriers and Facilitators for Medication Safety in Emergency Care Using a SEIPS Model: A Qualitative Study

**DOI:** 10.1155/jonm/3268082

**Published:** 2026-06-30

**Authors:** Ying Lau, Lilin Zhou, Yuen Man Chung, Hon Lon Tam, Sai Ho Wong

**Affiliations:** ^1^ The Nethersole School of Nursing, The Chinese University of Hong Kong, Hong Kong, China, cuhk.edu.hk; ^2^ Alice Ho Miu Ling Nethersole Hospital and Tai Po Hospital, Hospital Authority, Hong Kong, China, ha.org.hk; ^3^ Department of Nursing, Alexandra Hospital, Singapore, hosp-alexandra.gr

**Keywords:** barriers, designated checker, emergency department, facilitators, independent double-checking, thematic analysis

## Abstract

**Aim:**

The present study aimed to investigate the facilitators and barriers encountered by primary nurses and designated checkers in their participation with the designated independent double‐checking (IDC) process for the administration of high‐alert medications in the emergency department, employing the systems engineering initiative for patient safety (SEIPS) framework.

**Background:**

Designated IDC acts as a safety measure to prevent medication errors, provided by an experienced checker. However, the facilitators and barriers that influence this process remain unclear.

**Methods:**

An exploratory qualitative study was conducted using a purposive sample of 26 primary nurses and designated checkers. Data were collected through individual semistructured interviews and analysed using Braun and Clarke’s six phases of thematic analysis.

**Results:**

Our analysis revealed 15 facilitators and 16 barriers, which were classified according to the SEIPS domains: environment, organisation, people, task, tools and technology, process and outcome.

**Conclusion:**

The findings concerning the facilitators and barriers to implementing a designated IDC are a vital initial step in developing evidence‐based interventions to enhance medication safety.

**Implications for Nursing Management:**

The findings may suggest the maintenance of clear documentation, the promotion of effective communication, the conduct of regular audits, and the incorporation of IDC training into both orientation programmes and in‐service training, which is especially crucial for junior staff. These factors guide policymakers in restructuring the environmental layout, standardising IDC guidelines, ensuring sufficient staffing, fostering a nonhierarchical atmosphere, and promoting the adoption of technology.

## 1. Introduction

Medication errors can lead to serious adverse events, prolonged hospitalisation, extra medical treatment, morbidity, and death [1, 2]. Medication safety is a critical component of a functional and successful healthcare system [3]. A systematic review suggests that the pooled prevalence of medication errors in emergency department (ED) was 22.6%, based on findings from 24 articles across 12 countries [4]. The ED is a place of extreme vulnerability to the risks of medication errors [5]. A study indicated that there were 250 serious medication errors reported across 101 hospitals in the United States from 2011 to 2020 [6]. Another study revealed significant variability in healthcare professionals’ perceptions of patient safety within the ED from 24 European countries [7]. However, insufficient training, understaffing, challenges in managing patient flow, and overcrowding are the common problems [7]. In Hong Kong, a special administrative region of China (SAR), the Hospital Authority (HA) annually records the rates of dispensing errors as well as significant medication errors [8]. According to the 2025 annual report, during the first nine months of that year, there were 58.6 million medication items recorded, with the number of reported medication incidents reaching 8.9 million, including serious adverse events [9].

An independent double‐check (IDC) serves as a prudent safety measure aimed at reducing the risk of medication errors [10, 11]. The advantages of the independent verification are to prevent errors, reduce the chance of confirmation bias (where one person simply agrees with another without checking thoroughly), and encourage teamwork and shared responsibility [12–14]. Given that evidence reveals that IDCs can decrease medication errors [15], the HA recommends implementing IDCs in the EDs [16]. However, the guidelines provide limited details regarding the specific qualifications and work experience needed for checkers involved in the IDC process [16, 17]. One hospital proposed a designated IDC for high‐alert medication in its ED to mitigate the risk of medication errors [18]. The designated IDC pertains to the IDC process that requires a senior nurse checker. This checker must be a senior nurse with a minimum of 5 years’ experience in the ED and must have successfully completed structured IDC training. The designated IDC process involves two qualified nurses (primary nurse and designated checker) independently verifying a medication before giving it to patients, and the designated checker is formally assigned by the department to ensure accountability.

The designated IDC was intended to offer an additional safeguard for medication safety in EDs [18, 19]. Although this ED has launched the designated IDC system since Oct 2023, factors could be influencing the implementation of the IDC process, particularly regarding facilitators and barriers. By exploring these facilitators and barriers, we aim to ensure that the designated IDC is not simply a policy or guideline but a practical approach that can effectively function within the chaotic environment of the EDs [1]. Mapping these facilitators and barriers allows hospitals to develop evidence‐based interventions that are both practical and sustainable, ultimately enhancing patient safety outcomes [1]. The designated IDC in this hospital was operating as a pilot site, with intentions to implement the new practice in other hospitals. Our research team discovered that the IDC using designated checkers was more likely to be linked to full IDC compliance than the IDC utilising random checkers in our quantitative study [20]. Therefore, these practices may be deemed transferable to other EDs, both at local and global levels.

We identified several studies investigating factors associated with the administration of generic medications in various settings, including mental health care units, inpatient acute care units, EDs, and both adult and neonatal intensive care units (ICUs) in the United States of America [21], the United Kingdom [22], and Australia [23]. None of them paid attention to the process of IDC, especially in the ED. We found three studies that investigate nurses’ experiences with the double‐checking process in ICUs, internal medicine, and surgery units located in Norway [24], the Netherlands [25], and London, United Kingdom [14]. However, two studies [24, 25] did not specify the double‐checking employed, and only one study [14] concentrated on IDC. Hence, there is a deficiency of information in the existing evidence concerning the facilitators and barriers associated with the designated IDC process in the ED.

**FIGURE 1:**
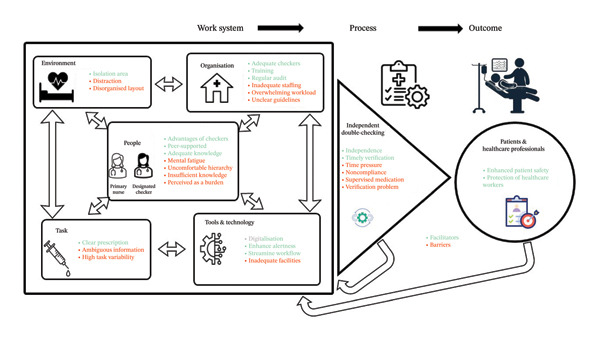
Systems engineering initiative for patient safety (SEIPS) model for elaborating a designated independent double‐checking of high‐alert medication administration in the emergency department.

Our research study adopted the systems engineering initiative for patient safety (SEIPS) model as a theoretical framework to explore the facilitators and barriers of the IDC process in the ED [26]. The SEIPS model is a human factor–based framework that views healthcare as a complex sociotechnical system, linking the design of work systems to processes and ultimately to patient and staff outcomes. It includes three interconnected components: the work system, processes, and outcomes (Figure 1). People, environment, tools and technology, tasks and organisation combine to form the work system, which, in turn, produces performance [26]. The environment refers to physical layout, location and factors like noise, location and lighting. The social–organisational environment describes ED in terms of structure, procedure, and organisational culture. The IDC process takes into account the external environment, such as the guidelines and regulatory context [26]. The process is how nurses carry out the IDC and its flows. The process is based on the physical, cognitive, and social–behavioural aspects of the nurses in the ED [26]. The outcome refers to the patients and healthcare professionals obtained from the work system and the IDC process [26]. The SEIPS offered guidance for the creation of semistructured questions (Table 1) and provided data analysis for the exploration of facilitators and barriers, thereby ensuring alignment with our research purpose. The present study aimed to explore the facilitators and barriers faced by primary nurses and designated checkers in their involvement with the designated IDC for the administration of high‐alert medications in the ED, utilising the SEIPS framework.

**TABLE 1:** Semistructured interview guide.

**Types**		**Questions**

Warm‐up questions		What are your views on the use of the designated checker system in the emergency department (ED) of your hospital?
		In your opinion, what are the factors that affect the independent double‐check (IDC) for high‐alert medication with a designated checker?

*SEIPS domains*		
Exploratory questions	Organisation	1. How does leadership affect the designated IDC for the high‐alert medication administration process?
		2. Tell me about how policy affects the IDC process in the ED.
		3. Please describe how culture affects the IDC medication administration.
		4. What types of training does your organisation offer for learning the IDC with a designated checker?
	Environment	5. How does the layout of the environment facilitate the IDC process in your department?
		6. Please share with me how the physical environment influences the IDC process.
	Task	7. How does clinical procedures influence the IDC process?
		8. Can you explain how the system for delivering medication functions within the IDC process?
	Tools & technology	9. Please explain how your current electronic system affects the IDC process.
		10. Can you provide your insights regarding the availability of equipment or facilities to support the IDC process?
	People	11. How do you feel in your role as a primary nurse or designated checker?
		12. Please explain how personal factors influence your role.
		13. How familiar are you, as a primary nurse or designated checker, with your delivery of the IDC process?
	Process	14. What is your experience of implementing the IDC for high‐alert medication?
		15. How do various factors impact you during the IDC process in your department?
	Outcome	16. What outcomes do you anticipate following the designated IDC process?
		17. How can these outcomes be beneficial for patient outcomes?
		18. Please share your perspective on the IDC process from the viewpoint of healthcare professionals or organisations.

Probe question		Can you tell me more?
		Can you elaborate on this answer more?
		Could you provide specific examples?

Closing Questions		Is there anything you’d like to add that wasn’t covered in this interview?
		Please tell me about any other thoughts you haven’t expressed above.
		Finally, are you okay with me contacting you for some follow‐up questions?

*Note:* SEIPS: system engineering initiative for patient safety model.

Abbreviation: IDC, independent double‐check.

## 2. Methods

### 2.1. Design and Setting

An exploratory qualitative study was adopted. The study was conducted in the EDs of a university‐affiliated hospital. The ED recorded approximately 157,000 attendances each day, translating to an average of 430 to 450 patients daily. The services provided encompassed immediate life support, trauma care, urgent medical care, triage systems, an observation ward, a gatekeeping role, and specialist support. The department employed roughly 200 to 250 nurses across different shifts. The present study was reported in accordance with the consolidated criteria for reporting qualitative research (COREQ) checklist [27].

### 2.2. Participants

A purposive sampling strategy was employed to recruit nurses of varying ranking from the ED, with the aim of capturing diverse perspectives by including both designated checkers and primary nurses. The inclusion criterion stipulated that participants must be registered nurses (RNs) who regularly administer high‐alert medication in the ED that requires an IDC process; physicians, students, and temporary staff were excluded.

### 2.3. Data Collection

Data collection was conducted from February 12, 2025, to May 21, 2025. We promoted the study by conducting an introductory session during the handover period and displaying a poster on the notice board in the ED. A research assistant invited eligible participants during their shift and explained the aim and nature of the study, along with the information sheet, to obtain a written consent form. The interview guide comprised open‐ended warm‐up, exploratory, probing, and closing questions. Furthermore, field notes were recorded for contextual detail and nonverbal cues. We improved the interview guide for clarity after distributing it to every member of the study team. To make sure the nurses could comprehend the interview instructions, we conducted two pilot interviews prior to data collection.

Before the interview, we gathered demographic and professional characteristics for each participant, including gender, age, position, educational level, nursing experience, and ED experience. An individual interview was scheduled at a suitable time during the participants’ shift, depending on the clinical workload. One female research assistant with a bachelor’s degree finished 5‐day qualitative study training by a female qualitative expert with a Doctor of Philosophy. The trained female research assistant conducted face‐to‐face interviews in Cantonese, taking place in a private room within the department. Individual interviews were conducted until there were no new themes emerging from the participants. The saturation was reached with the 15th designated checkers and the 11th primary nurses. Each interview lasted between 28 and 50 min. Upon completion, participants received a small gift (HK$50 ≈ UD$6.41) as a token of appreciation for their time.

### 2.4. Data Analysis

All audio recordings were transcribed verbatim and anonymised. We uploaded and organised the transcripts using NVivo Version 1.7.1 [28]. Two researchers (Ying Lau and Lilin Zhou) independently analysed the qualitative data. A deductive thematic analysis utilising the six‐phase framework established by Braun and Clarke [29] and guided by the SEIPS theoretical model [26]. The analytical framework comprised the components of environment, organisation, people, tools and technology, task, process and outcome. First, we immersed ourselves in the data by thoroughly reading the transcripts and field notes. Second, we systematically coded intriguing findings across the dataset, identifying patterns and insights that emerged from the data analysis process, which were based on the components of the SEIPS model. Third, we grouped the codes into themes to interpret their meaning. Fourth, we refined and verified the themes against the datasets. Fifth, we assigned each theme a concise and descriptive name. Finally, we produced a report to present coherent findings in relation to the research question. The quotes were translated into English by a researcher who was fluent in both Cantonese and English, and then, they were interpreted back into Cantonese by another bilingual researcher to make sure they matched the original transcripts [30]. We developed a codebook following multiple discussions within the research team. A total of 54 codes were extracted from the transcription. The thematic analysis revealed 15 facilitators and 16 barriers encountered during the application of IDC through the designated checker system with codes and illustrative quotations, as shown in Supporting Files 1 and 2.

### 2.5. Rigour

To ensure the trustworthiness of this qualitative study, multiple strategies were employed throughout the research process, aligned with the criteria established by Lincoln and Guba [31] for credibility, dependability, transferability and confirmability. Credibility was enhanced by purposively sampling a diverse range of participants, ensuring representation across roles, age ranges and clinical experience. The analysis process involved two researchers who independently coded the data. They then engaged in discussion to compare, discuss and reach a consensus on the codes and emerging themes, thereby refining the interpretive framework. Dependability was addressed by maintaining a comprehensive audit trail of all analytical decisions, which provides a transparent record. Transferability was enhanced by offering detailed, contextual descriptions of the study setting, participant characteristics and data collection methods. This allowed readers to evaluate how the findings might apply to other similar contexts. Confirmability was sought from both the perspectives of primary nurses and designated checkers to triangulate the findings, and detailed records of data analysis were kept tracing the research process.

### 2.6. Ethical Considerations

The research was conducted in full accordance with the ethical principles outlined in the Declaration of Helsinki [32]. Ethical approvals for this study were reviewed and approved by the Joint Chinese University of Hong Kong‐New Territories East Cluster Clinical Research Ethics Committee for the Chinese University of Hong Kong (CREC Ref. No.: 2024.416) and Prince of Wales Hospital (CREC Ref. No.: 2024.415) prior to commencement. Written informed consent was obtained from all participants. The consent procedure included a clear explanation of the study’s purpose, the voluntary nature of participation, the agreement to audio‐record the interviews and the assurance that all collected data would be treated with strict confidentiality. Participants were explicitly informed of their right to withdraw from the study at any time without consequence.

## 3. Results

A total of 26 nurses participated in the study. Their demographic and professional characteristics are summarised in Table 2. The sample comprised 10 male and 16 female participants. Among the 15 designated checkers (D) interviewed, with different rankings, were 6 advanced practice nurses, an associate nurse consultant, a RN (specialty), and 7 RNs. The age of designated checkers fell within the range of 25 to 60 years, and their experience in the ED varied from 5 to 13 years. All 11 primary nurses (P) interviewed were RNs. Their ages ranged from 20 to 34 years, with ED experience spanning from < 1 year to 4 years.

**TABLE 2:** Participant characteristics (*N* = 26).

Id	Gender	Age	Position	Education level	Nursing experience	ED experience	Duration
*Designated checkers (n = 15)*							
D1	Male	31–34	Advanced practice nurse	Master’s degree	> 9–10 years	8 years	34 min
D2	Male	35–40	Advanced practice nurse	Master’s degree	> 10–15 years	8 years	40 min
D3	Female	51–55	Advanced practice nurse	Master’s degree	> 20–30 years	12 years	28 min
D4	Male	31–34	Advanced practice nurse	Bachelor’s degree	> 9–10 years	7 years	34 min
D5	Male	41–44	Associate nurse consultant	Master’s degree	> 15–20 years	11 years	42 min
D6	Female	31–34	Registered nurse	Bachelor’s degree	> 5–6 years	4 years	28 min
D7	Female	31–34	Registered nurse	Master’s degree	> 9–10 years	6 years	50 min
D8	Female	25–30	Registered nurse	Bachelor’s degree	> 6–7 years	5 years	35 min
D9	Female	45–50	Registered nurse	Master’s degree	> 20–30 years	< 1 year	36 min
D10	Female	35–40	Advanced practice nurse	Bachelor’s degree	> 15–20 years	11 years	35 min
D11	Female	55–60	Registered nurse	Bachelor’s degree	> 30 years	13 years	42 min
D12	Female	45–50	Registered nurse	Diploma	> 20–30 years	10 years	40 min
D13	Female	31–34	Registered nurse (specialty)	Bachelor’s degree	> 9–10 years	8 years	31 min
D14	Female	25–30	Registered nurse	Bachelor’s degree	> 5–6 years	4 years	35 min
D15	Male	35–40	Advanced practice nurse	Master’s degree	> 10–15 years	9 years	29 min

*Primary nurses (n = 11)*							
P1	Female	25–30	Registered nurse	Bachelor’s degree	> 3–4 years	2 years	31 min
P2	Female	31–34	Registered nurse	Bachelor’s degree	> 10–15 years	4 years	28 min
P3	Female	25–30	Registered nurse	Bachelor’s degree	1–2 years	2 years	28 min
P4	Female	25–30	Registered nurse	Bachelor’s degree	> 4–5 years	2 years	39 min
P5	Male	31–34	Registered nurse	Bachelor’s degree	> 3–4 years	2 years	27 min
P6	Female	25–30	Registered nurse	Master’s degree	1–2 years	2 years	35 min
P7	Male	25–30	Registered nurse	Bachelor’s degree	> 2–3 years	< 1 year	28 min
P8	Male	25–30	Registered nurse	Bachelor’s degree	> 2–3 years	< 1 year	33 min
P9	Male	25–30	Registered nurse	Bachelor’s degree	> 4–5 years	4 years	35 min
P10	Female	20–24	Registered nurse	Bachelor’s degree	1–2 years	2 years	35 min
P11	Male	25–30	Registered nurse	Bachelor’s degree	< 1 year	< 1 year	34 min

*Note:* D = designated checker; mins = minutes; P = primary nurse.

Abbreviation: ED, emergency department.

**FIGURE 2:**
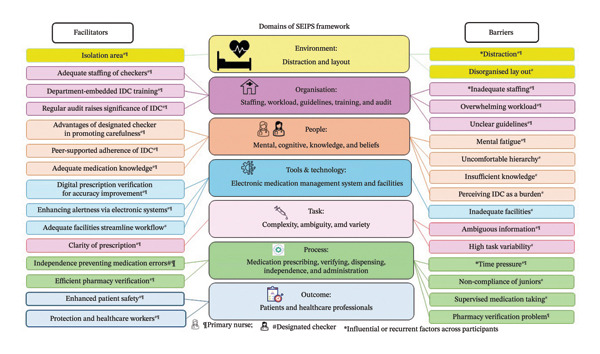
Facilitators and barriers for designated independent double‐checking of high‐alert medication administration in the emergency department using a SEIPS framework.

The thematic analysis yielded a total of 15 facilitators and 16 barriers for designated IDC of high‐alert medication administration in the ED based on the domains of the SEIPS framework. The main results are shown in Figure 2.

### 3.1. Environment

Distractions and the environmental layout were identified as both facilitators and barriers to the designated IDC of administering high‐alert medication in the ED. These factors can significantly influence the efficiency and accuracy of medication delivery. Most of the participants expressed that distraction was the main barrier. A primary nurse highlights the importance of a distraction‐free area for the IDC process as follows: *“So, ideally, during the IDC process, we would hope to administer medications in an environment that is relatively quiet or more secluded” (P3, Female)*. A designated nurse also explained that sudden interruptions could disrupt their focus during the checking process. *“Most of the time, there are numerous interruptions during the IDC process, such as the patient’s family, the patient themselves, and our colleagues” (D5, Male).* Another designated nurse expressed her difficulty in locating the patient due to the highly mobile nature of the ED. *“Patients in the ED are highly mobile, making it time-consuming to locate them, especially if they have been in x-ray, CT, or other areas” (D7, Female)*.

### 3.2. Organisation

Organisational factors—including staffing, workload, guidelines, training and audits—were linked to both facilitators and barriers. More than half of the participants expressed concerns about the inadequate staffing of designated nurses during night shifts. Both the designated nurse and primary nurse voiced *“Having more staff available to act as designated checkers at the hospital and department level would make it easier to find and access medications during administration” (P6, Female)*. *“It is often challenging to locate a designated checker to administer IDC medications, particularly during night shifts” (D13, Female)*. Participants noted that the significant workload and unclear guidelines create challenges for implementing IDC. *“Sometimes, the guidelines lack clarity regarding which medications require double-checking and which do not” (D9, Female)*. They believed that simulated training and regular audits enhanced their skills and increased their awareness of the IDC process. *“Actually, I feel that learning in a simulation environment might be safer” (D5, Male)*. *“In my opinion, conducting audits periodically is also beneficial” (P7, Male)*.

### 3.3. People

For personal factors, our findings included aspects such as mental state, cognitive abilities, knowledge and beliefs. Participants valued having a designated checker for IDC and acknowledged the necessity of strictly following the steps. *“I believe this process is beneficial because it can help reduce the likelihood of medication errors” (P8, Male)*. *“I believe this checking system functions effectively to adhere to the IDC procedure correctly” (D10, Female)*. A designated checker noted that the IDC process encourages meticulous checking, while a primary nurse emphasised the importance of understanding medication knowledge. *“This is especially important if we strictly adhere to the IDC procedures” (D15, Male)*. *“IDC requires both parties to have a very clear understanding of the prescription and to be highly familiar with the medication preparation itself” (P1, Female)*.

Participants identify several personal factors as barriers, including mental fatigue, uncomfortable hierarchy, insufficient knowledge and the perception of IDC as a burden. Here is a selected voice from a participant: *“I often feel physically fatigued, and mentally, I struggle with alertness and concentration. We need to find an additional person to double-check the medication, which creates a certain burden” (D5, Male)*.

### 3.4. Tools and Technology

For factors related to tools and technology, the findings included electronic medication management and associated facilities. The participants perceive the digitalisation of medication administration as a key facilitator in the IDC process, which uses electronic systems and scannable codes for enhanced verification accuracy, improved readability and increased alertness. Some participants offered the following comments: *“IPMOE (Inpatient Medication Order Entry, a computerised medication prescribing, dispensing, and administering system) indicates whether the verification was completed by two individuals… the process does help to improve safety. (P9, Male)*. *“Scanning QR codes would facilitate the IDC” (D13, Female)*. On the other hand, two designated checkers expressed that the inadequate facilities, along with setting restrictions, posed significant barriers to effectively carrying out the IDC process. *“The situation becomes quite challenging due to the less-than-ideal environment” (D7, Female)*. *“We often find ourselves holding the patient’s files while carrying medications in a paper cup” (D10, Female)*.

### 3.5. Task

Our findings indicate that the implementation of the IDC encountered several facilitators and barriers to the task, including clear prescription, ambiguous information and high task variability. Both the designated checker and primary nurse mentioned the importance of clear orders during the IDC process as follows: *“I wish for clearer prescriptions to reduce the likelihood of errors” (D3, Female)*. *“I believe the most crucial factor is ensuring that prescriptions are easily readable and clear, whether entered via IPMOE or recorded in the treatment sheet within the resuscitation room” (P6, Female)*. The participants were concerned about ambiguous information and frequent changes of prescription. *“Sometimes, doctors mention only the name of a medication without detailing the dosage, which could affect IDC” (P2, Female). “Doctors’ decisions can change swiftly. Infusion rates are frequently adjusted, and occasionally a medication is prescribed; however, before administration, it may be altered to an entirely different medication” (D7, Female).*

### 3.6. Process

Our findings regarding the facilitators encountered during the IDC process encompassed independence preventing medication errors and efficient pharmacy verification. Both designated checkers and primary nurses appreciated the importance of independence in enhancing accuracy and reducing medication errors. *“If we can effectively implement an IDC in an independent manner, it should improve overall safety levels” (D4, Male)*. *“Having a second individual independently check the work helps minimise the risk of medication errors” (P5, Male)*. However, participants identified several barriers they encountered during the IDC process, including time pressure, noncompliance of juniors, the need for supervised medication taking and issues related to delayed pharmacy verification. Among the various barriers identified, many participants cited time pressure as a prevalent issue. Some concerns are highlighted as follows: *“Doctors often provide verbal prescriptions rapidly, … I may not be entirely sure whether the dosage I remember is accurate” (P10, Female)*. *“I have noticed that some junior staff occasionally do not adhere fully to the IDC steps and procedures” (D10, Female)*. Two participants expressed that *“In paediatric cases, we may not be able to administer the medication directly to the child… Instead, we might provide the medication to the parent for them to administer to the child” (D7, Female). “Sometimes, when we call the pharmacy to ask them to verify the medication, it can take ten or even 20 min” (P4, Female)*.

### 3.7. Outcome

The participants provide commentary on the outcomes of the designated IDC for both patients and healthcare professionals. *“I view IDC as a safeguard for patients. If one nurse makes an error, the other nurse should be able to identify it” (P4, Female)*. *“I think that from a coworker’s point of view, there is a protective element. Someone with more experience should be the designated checker because they are more likely to know more about medications and be able to notice more details” (D7, Female)*.

## 4. Discussion

We recruited 15 designated checkers and 11 primary nurses to identify the facilitators and barriers encountered during the designated IDC of high‐alert medication administration in the ED. Following the components of the SEIPS framework, 15 facilitators and 16 barriers were identified.

### 4.1. Facilitators

We found that establishing a distraction‐free zone in the ED would facilitate the designated IDC. This finding is consistent with a prior study conducted in adult and neonatal ICUs [23], which pointed out the importance of a quiet, interruption‐free environment in facilitating safe medication administration. Both the ED and ICU play vital roles in the IDC of high‐alert medications due to the inherently high‐stress and fast‐paced nature of these environments, which are often subject to interruptions [33]. Establishing a quiet zone enables staff to concentrate fully on the IDC process, minimising cognitive overload and preserving the integrity of this crucial safety measure [34].

Our study found that having adequate staffing for designated checkers is a key facilitator that aligns with previous research in adult and neonatal ICUs [23]. Both EDs and ICUs require rapid decision‐making, manage high patient acuity and utilise complex drug regimens. Therefore, enhancing staffing levels in these high‐stress environments could improve medication safety and reduce the likelihood of missed checks [33]. Adequate staffing allows for greater flexibility, enabling more emergency nurses to address urgent needs. Meanwhile, the primary nurse and designated checker can perform the double‐check independently [13]. Aligning with the recommendations of the Institute for Safe Medication Practice [35], the department should embed designated IDC training. The training could be integrated into an orientation program for new staff as well as ongoing in‐service training. It may also utilise a simulation environment to foster a safe learning atmosphere [36]. In addition, we found that regular audits raised the significance of IDC for high‐alert medication. These audits ensured that staff actively engaged in monitoring safety practices rather than merely following a policy [35].

Our findings showed that participants valued the advantages of designated checkers because designated checkers were trained with rich experience and specialised skills, which is crucial for ensuring patient safety during critical situations [19]. Peer support can facilitate adherence to the IDC process. When peers actively support one another, the responsibility for accurate checking is shared collectively rather than placed solely on one individual [12]. Our study found that the IDC process induces carefulness of checking, as this pause allows participants to consciously reassess details such as dose, route and patient identity [34]. Furthermore, adequate medication knowledge is a facilitator that is consistent with a prior study conducted in an inpatient acute care unit and the ED of an American hospital [21]. It is because both settings have more high‐alert medications compared to the generic units, and knowledge could enhance staff confidence, error detection and adherence to safety procedures [37]. Nurses with medication knowledge may process information more efficiently, allowing them more mental bandwidth for thorough checking [10].

Our study found that information technology (IT) can facilitate the administration of high‐alert medications. This includes how digitalisation prevents medication errors, enhances alertness through electronic systems, and improves clarity of prescription with digital systems. The findings are supported by a previous study conducted in an adult ICU [14], which suggested that an IT system could strengthen the IDC process by minimising human error, facilitating rapid verification and reducing cognitive load through electronic tools in both settings, such as the IPMOE system and barcode scanning [38]. The participants suggested that providing adequate facilities, such as a dedicated electronic set for medication administration for each nurse, can help streamline workflow and reduce delays caused by a lack of devices.

The IPMOE system integrates prescribing, dispensing and administration of medications. In the ED, it can significantly streamline the identification, dispensing and confirmation during the IDC process for medication safety by embedding technology into each stage of the workflow [39]. During the prescribing stage, the system diminishes variability and errors associated with prescriptions. The integration of barcode‐assisted medication administration and automated pharmacy systems propels hospitals towards a closed‐loop medication management system [38]. Consequently, barcode verification ensures that the drug dispensed aligns with the electronic order [39].

The IPMOE system enforces IDC by necessitating two staff logins at the dispensing stage. Additionally, it requires a second nurse to electronically validate high‐alert medications prior to administration during the administration stage. Bedside barcode scanning can confirm the patient’s identity and verify that the medication matches. This would enable IDC to be conducted promptly, even in emergency situations [10]. The participants acknowledged the necessity of automatic IDC alerts within the electronic system, and each alert could enhance accountability and traceability for safety audits [38]. This system automates part of the double verification process, which reduces reliance on memory and manual cross‐checks [39].

In our study, clarity of prescription was a facilitator, where clear prescriptions might reduce ambiguity, minimise cognitive load and ensure both parties were verifying the same accurate information [40]. The nature of independence as a facilitator has been shown to strengthen accuracy and prevent medication errors. When each nurse verifies the medication order, patient chart, and calculations independently, they do so without influence from one another [34]. This approach helps prevent the repetition of the same error [40]. Furthermore, independence may increase vigilance and reduce cognitive biases, such as confirmation bias [10].

To achieve genuine independence for IDC in a chaotic ED, it is essential to implement both structural safeguards and behavioural reinforcements [33]. The ED should facilitate the physical separation of tasks by assigning distinct staff members to prepare and check medications in separate areas, thereby minimising unconscious influence [14, 34]. Nursing managers ought to establish a clear protocol accompanied by an IDC checklist to explicitly define IDC responsibilities and steps, ensuring that each checker understands their role throughout the IDC process [12]. However, in scenarios where independence may be compromised, staff should have a straightforward method to flag and postpone administration until proper verification can be conducted [15].

However, efficient pharmacy verification is crucial, as pharmacists are responsible for ensuring that prescriptions are appropriate, correctly dosed and free from potential drug interactions. This verification must be conducted swiftly to allow the IDC to proceed without delay [41]. The participants believed that the IDC could enhance patient safety, as both engaged in deliberate and mindful verification [10]. In addition, primary nurses were better protected when a designated checker was present, which is consistent with a prior study [24]. The IDC process mitigates professional risk, as medication errors can lead to serious legal and disciplinary consequences [12].

### 4.2. Barriers

The findings align with those of previous studies conducted in the inpatient acute care unit, ED and mental health unit [21, 22], where distractions and an overwhelming workload were identified as barriers. Distraction is the most influential barrier among the participants. A possible explanation is that staff in these settings experience high multitasking overload and frequent interruptions, which may diminish their ability to maintain vigilance and focus on primary tasks, thereby increasing the risk of medication errors [42]. Frequent interruptions could divert focus from the medication task, and a heavy workload in the ED forces nurses to prioritise speed over thoroughness [35, 43]. The nursing managers can designate quiet zones for medication preparation and checking, thereby minimising environmental noise and interruptions from other clinical tasks [34]. Furthermore, allocating specific staff members for IDC during each shift can help reduce the disruptions caused by multitasking [44]. Additionally, well‐structured in‐service training programmes tailored to chaotic ED scenarios could enhance the resilience of ED staff, enabling them to manage interruptions effectively during the IDC process [45].

The disorganised layout hindered the ability to locate patients in the ED, and this finding aligns with geographic factors noted in a previous study, which was conducted in the adult ICU [14]. Poor physical layouts in the ICU [46] and the ED [47] significantly contribute to nonadherence to double‐checking protocols, as the lack of space renders verification impractical [48]. The ED layout could directly influence workflow efficiency and safety outcomes, with poor design increasing risks of error [47].

Our study found inadequate staffing to be a barrier, particularly during night shifts. The barrier occurred recurrently among the participants. This observation aligns with the results of previous studies conducted in the ICU, internal medicine and surgery units [24, 25]. During night shifts, staffing ratios in these units are low, which makes it challenging to locate a second checker to verify high‐alert medications [48]. The IDC is labour‐intensive, and insufficient staffing results in delays, skipped checks or superficial compliance [33]. We also found that unclear guidelines create a barrier to IDC, which is consistent with a prior study in the ICU [24]. Hence, staff in both settings are unsure about who should perform the second check and under what circumstances it increases the likelihood of errors with high‐alert medications [14]. If the guidelines are vague or inconsistent, nurses may interpret them differently, leading to variability in practice and reduced reliability [34], which can ultimately affect patient outcomes and the overall effectiveness of the IDC process.

Our findings showed that mental fatigue and insufficient knowledge were important barriers, particularly among junior nurses. These results are similar to the phenomenon in previous studies in mental health units and ICUs [14, 22]. Junior nurses may lack a comprehensive understanding of drug mechanics, dosages or associated risks, which can hinder their capacity to identify and challenge potential errors [14]. Moreover, they may find it challenging to interpret or consistently apply medication policies, particularly when the guidelines are complex [22]. Mental fatigue can impair cognitive performance, memory recall and vigilance, resulting in staff potentially overlooking crucial details [14]. A lack of pharmacological knowledge or unfamiliarity with the IDC process may hinder their ability to critically assess medication details [12].

Additionally, we found that an uncomfortable hierarchy is a barrier. The junior staff might be reluctant to question senior colleagues because of hierarchical pressures, thereby undermining independence [25]. Hence, a standardised protocol is essential to establish IDC as a shared task rather than a hierarchical challenge [34]. The department should highlight that IDC is a safety partnership and foster a culture that encourages speaking up, framing questions as a means of protecting patients instead of undermining colleagues [33]. Additionally, rotating IDC pairs can help prevent repetitive junior–senior dynamics, thereby normalising mutual checks [49].

Some participants viewed the IDC process as a burden, which aligns with prior studies in ICUs [14, 24]. Both EDs and ICUs operate in fast‐paced environments characterised by urgent interventions, which complicate the IDC process [14]. Therefore, staff may view IDC as unnecessary and can foster resistance, ultimately leading to reduced compliance [24]. These attitudes may also lead to a diminished appreciation for safety practices [14]. The nursing managers could embed the IDC process into the workflow in ways that minimise disruption and make IDC a routine safety step rather than an extra burden [34]. The department should share data showing how IDC prevents medication errors [33] and celebrates successful catches to reinforce its values and motivate staff [34]. We identified inadequate facilities as a barrier. The less‐than‐ideal environment and restricted setting may lead staff to variability and reduced adherence to the IDC steps [14, 50]. Inadequate facilities impede efficient retrieval and verification processes, thereby increasing the cognitive load on staff [40]. This situation results in frustration and reinforces the perception that the IDC is impractical in urgent care settings [12].

Additionally, ambiguous information can lead to different interpretations, resulting in inconsistencies in task execution and potentially compromising patient safety [33]. Consistent with previous research conducted in the ICU and the mental health unit [22, 24], verbal prescriptions issued in emergency situations present challenges for the IDC process due to uncertainties regarding their accuracy [12]. The high task variability was a barrier because of frequent changes in prescriptions. When orders change repeatedly, the “source of truth” becomes unclear [35]. The unclear communication serves as a barrier, aligning with a pattern observed in a prior study in a mental health unit, where participants misinterpreted or made assumptions about information [22]. The ED department could consider implementing structured communication tools such as closed‐loop communication and concise communication while avoiding jargon. Closed‐loop communication involves one staff member verbalising the information, followed by the other staff member repeating it back to confirm its accuracy [51]. The ED staff could utilise the situation, background, assessment, recommendation (SBAR) framework to facilitate clear and concise communication [52]. It is important to use standardised terminology instead of jargon to prevent misinterpretation [53].

Time pressure serves as a repeated barrier, aligning with a prior study conducted in the internal medicine, surgery and ICUs of a Dutch hospital [25]. Staff in these settings handle critical conditions requiring immediate intervention and believe that IDC may delay treatment [14]. The urgency of care conflicts with the deliberate, methodical nature of the checking process [40]. Under time pressure, nurses might bypass the IDC process or rush the verification [12, 25]. The simultaneous demands created challenges due to time pressure, and a previous study conducted in a mental health unit observed similar patterns [22]. Hence, the staff in ED may often be pulled into multiple urgent tasks simultaneously, which reduces the time and focus necessary for the IDC [34]. However, challenges may emerge from the complexities involved in patient‐related nursing care, particularly in emergency situations [54]. Consequently, the IDC process could be tailored according to the patient’s nursing risk profile, thereby integrating medication safety within a broader framework of proactive risk stratification and early detection of deterioration [55].

We observed barriers related to noncompliance among junior staff, specifically their failure to adhere to the IDC step and an overreliance on designated checkers. These results are consistent with findings from a previous study in the ICU [14], which suggested that junior staff perceive senior colleagues as being less prone to making mistakes and having reduced cognitive capacity to conduct a robust IDC effectively. These results highlight that simulation‐based training has the potential to enhance both confidence and accuracy in the IDC process among junior staff. This approach may reduce hierarchical dependence and encourage junior staff to take an active role in safety checks [33]. Nursing managers could foster a culture of shared accountability, emphasising the IDC as a mutual responsibility rather than a hierarchical task. This would empower junior nurses to engage in more active cognitive processing during the IDC process [56].

We found that supervised medication taking, particularly in some paediatric cases, presents a barrier because children often need assistance or require their parents to supervise. This adds complexity to the IDC process [50]. The ED staff could encourage parents to be active participants in the medication process by explaining the rationale for each drug and involving them in verification [57]. Structured training can enhance parental involvement in medication administration, according to the literature. This training should utilise visual aids, provide simplified instructions, and incorporate teach‐back methods to ensure that parents fully understand the information [53]. The pharmacy verification problem can act as a barrier. Delays, inconsistencies or a lack of clarity in pharmacy verification may have jeopardised the protections that the IDC provided [12]. The findings suggested the need for interdisciplinary collaboration through joint training sessions involving physicians, pharmacists and nurses [6]. This approach aims to foster a mutual understanding of workflow constraints and enhance the adequacy of pharmacy staff during peak hours in the ED to prevent bottlenecks [6]. Consequently, ED pharmacists could enhance the timeliness and accuracy of medication verification.

### 4.3. Strengths and Limitations

To the authors’ knowledge, this is the first qualitative study that investigates both the facilitators and barriers associated with designated IDC in the ED from the perspectives of primary nurses and designated checkers. We adopted a SEIPS model, which outlines the work system, process and outcome [26], as a framework for this study. This ensured that our data collection and analysis were systematically and theoretically aligned with the research objectives. Two independent researchers conducted qualitative data analysis using Braun and Clarke’s six phases of thematic analysis to minimise bias and enhance the validity of the interpretations. Additionally, by incorporating crucial elements like credibility, dependability, transferability and confirmability, this study guaranteed the rigour of the research. However, there are several limitations to this study. First, there is only one hospital, and the results might not reflect the full spectrum of opinions of nurses. Second, participants were volunteers, so their self‐selection may not represent the views of all potential participants. Third, the interview quotes were translated from Cantonese into English. Because of inherent linguistic and cultural variations, this method carries the danger of misinterpretation, which could affect how accurately the original language’s meaning is translated.

### 4.4. Recommendations for Further Research

Future research should replicate this study across various hospitals, both within different regions and internationally. This approach could reveal the contextual, cultural and organisational nuances that influence the practice of a designated IDC. Further investigation could explore the perspectives of policymakers, nurse managers, physicians and pharmacists, thereby enriching the IDC process with vital insights. Each group has distinct priorities and constraints that influence the implementation and sustainability of IDC, such as policymakers focussing on regulatory compliance, nurse managers prioritising patient care efficiency, physicians emphasising clinical outcomes and pharmacists addressing medication management. Future research ought to investigate whether certain challenges may also represent manifestations of complexity in patient‐related nursing [55]. Future exploration of the challenges faced during the IDC process may connect patient complexity, resource utilisation, workflow efficiency and quality of care [58]. Future research could strengthen its findings by employing a mixed‐methods approach, which would capture both quantitative measurement outcomes and qualitative human factors, thereby providing both evidence‐driven and context‐sensitive knowledge.

### 4.5. Implications for Nursing Management

Exploring facilitators and barriers associated with the designated IDC of high‐alert medications holds significant implications for nursing management, both from policy and practice perspectives. Considering that distractions, a lack of checkers at night and time pressure were the primary barriers identified by the participants, nursing managers should prioritise allocating resources to address these issues. The policy implications emphasise the need to restructure the layout of the environment to establish a distraction‐free area, standardise IDC guidelines by clearly delineating the types of medications and ensure sufficient staffing of designated checkers for the night shift. The IDC process could be customised to align with the nursing risk profile of the patient, considering the complexities related to their nursing needs [55].

In addition, encouraging a nonhierarchical atmosphere for open communication, and promoting the use of technology through automated alerts for high‐alert medications—all aimed at fostering a culture of medication safety. The practice implications, which include maintaining clear documentation, fostering effective communication and conducting regular audits, can enhance the IDC process within the ED. Nursing managers could incorporate standardised nursing terminologies and structured documentation systems into electronic health records to minimise ambiguity, enhance traceability and facilitate safer medication processes in complex ED settings [59]. Incorporating IDC training into orientation programmes and in‐service training is particularly important for junior staff to help them understand the importance of the IDC, pharmacological knowledge and the details of the IDC process. Furthermore, collaboration among various disciplines, including physicians and pharmacists, is essential for ensuring clarity in prescriptions and timely verification of medications.

## 5. Conclusion

Overall, this qualitative study explored the facilitators and barriers to implementing a designated IDC for high‐alert medication, as perceived by primary nurses and designated checkers in an ED. This is an initial step necessary for informing evidence‐based activities and recommendations to target these barriers. The findings illuminate the influential factors of distraction, inadequate checkers at night shift and time pressure, thereby assisting nurse managers in effective resource planning. Addressing these challenges through strategic design and focused training may enhance medication safety in high‐pressure situations. Future research has the potential to identify both patient‐level and system‐level facilitators and barriers that could enhance the alignment between policy and practice, as well as promote interprofessional collaboration.

## Author Contributions

Ying Lau: conceptualisation, methodology, validation, investigation, resources, data curation, writing–original draft, writing–review and editing, supervision, project administration and funding acquisition. Lilin Zhou: methodology, software, formal analysis, investigation, data curation, writing–original draft, writing–review and editing and visualisation. Yuen Man Chung: conceptualisation, writing–review and editing and supervision. Hon Lon Tam: conceptualisation, and writing–review and editing. Sai Ho Wong: validation, formal analysis and writing–review and editing.

## Funding

This study was funded by the Fund for Evidence‐based Practice Improvement Collaborative Projects by the Nethersole Evidence‐Based Nursing Practice Unit‐Nethersole Group Hospitals, Hong Kong SAR, China (Project code: 6906885, Grant Number: NEPNU_2024_LY).

## Disclosure

All authors have read and approved the final draft that was submitted for publication.

## Ethics Statement

Ethical approval was obtained by the Joint Chinese University of Hong Kong‐New Territories East Cluster Clinical Research Ethics Committee for the Chinese University of Hong Kong (CREC Ref. No.: 2024.416) and Prince of Wales Hospital (CREC Ref. No.: 2024.415).

## Conflicts of Interest

The authors declare no conflicts of interest.

## Supporting Information

Additional supporting information can be found online in the Supporting Information section.

## Supporting information


**Supporting Information 1** Supporting File 1: Facilitators, codes and illustrative quotations of a designated independent double‐checking process for high‐alert medication administration using a SEIPS framework. Supporting File 2: Barriers, codes and illustrative quotations of a designated of a designated independent double‐checking process for high‐alert medication administration using a SEIPS framework.

**Supporting Information 2** COREQ checklist: Consolidated criteria for reporting qualitative studies (COREQ): 32‐item checklist.

## Data Availability

The datasets generated and analysed during this study are not publicly available to safeguard their use in planned future research. Access may be granted upon reasonable request to the corresponding author.
