# Supplementary material for: Barriers and Facilitators for Medication Safety in Emergency Care Using a SEIPS Model: A Qualitative Study
Source: J Nurs Manag. 2026 Jun 30;2026:3268082. doi: 10.1155/jonm/3268082 (PMC13317145; doi:10.1155/jonm/3268082)
Supplement: Supplementary file 1 — Supporting Information 1 Supporting File 1: Facilitators, codes and illustrative quotations of a designated independent double‐checking process for high‐alert medication administration using a SEIPS framework. Supporting File 2: Barriers, codes and illustrative quotations of a designated of a designated independent double‐checking process for high‐alert medication administration using a SEIPS framework. [file JONM-2026-3268082-s001.docx]

Supplementary File 1: Facilitators, codes, and illustrative quotations of a designated independent double-checking process for high-alert medication administration using a SEIPS framework.

| Domains of SEIPS framework | Facilitators | Codes | Illustrative quotations |
| --- | --- | --- | --- |
| Environment | Isolation area^#¶^ | Quiet and isolated environment | (IDC should be) In a very quiet and isolated environment, free from other distractions… In that situation, I can really dedicate myself to focusing on the checks. (D2, Male, Designated checker, P6 Lines 184-188) |
|  |  |  | So, ideally, during the IDC process, we would hope to administer medications in an environment that is relatively quiet or more secluded. (P3, Female, Primary checker, P4 Lines 98-99) |
| Organisation | Adequate staffing of checkers^#¶^ | Suggestion of additional checkers | I believe that training additional colleagues to become designated checkers would be beneficial. This would help minimise instances where it takes a long time to locate another checker. (D6, Female, Designated checker, P4 Lines 100-101) |
|  |  |  | Having more staff available to act as designated checkers at the hospital and department level would make it easier to find and access medications during administration. (P6, Female, Primary checker, P6 Lines 162-163) |
|  |  | Sufficient checkers each shift | I would like to have more designated checkers on each shift; it would enhance convenience for everyone, eliminating the need to spend time searching for one. (D8, Female, Designated checker, P6 Lines 155-156) |
|  |  |  | When organising the duty roster, it’s essential to ensure that during each shift, a minimum of one-third, or even half, of the staff members are assigned as checkers. (P6, Female, Primary checker, P4 Lines 117-119) |
|  | Department‑embedded IDC training^#¶^ | Orientation programme including IDC | I firmly believe that junior staff receive training before working in the resuscitation room. They undergo two days of classroom instruction, followed by a week of shadowing an APN (advanced practice nurse). (D12, Female, Designated checker, P7 Lines 187-190) |
|  |  |  | Our ward manager plays a demonstration video in which they personally show how to carry out IDC properly. (P11, Male, Primary checker, P2 Lines 47-48) |
|  |  | Simulated training for IDC | Actually, I feel that learning in a simulation environment might be safer… I think it would be helpful to recreate some real cases of errors using simulation. (D5, Male, Designated Checker, P3 Lines 68-75) |
|  |  |  | Sometimes, a colleague who often works in the high-dependency unit may not come across medications that need a designated checker. In such instances, simulation training could be beneficial. (P10, Female, Primary checker, P1 Lines 25-29) |
|  | Regular audit raises significance of IDC^#¶^ | Need occasional audits and attention | We have watched a video, and there are occasional audits. I find occasional audits beneficial, as they serve as reminders of any gaps or overlooked elements. (D13, Female, Designated checker, P2 Lines 34-36) |
|  |  |  | I believe it is important for everyone to have a clear understanding of IDC and to be aware of which drugs need special attention. In my opinion, conducting audits periodically is also beneficial. (P7, Male, Primary checker, P1 Lines 19-20) |
| People | Advantages of designated checker in promoting carefulness#¶ | Benefits of designated checker¶ | When a designated checker is present, I believe the IDC is more capable of adhering to its principles. (D12, Female, Designated checker, P2 Lines 35-37) |
|  |  |  | IDC involves a more experienced colleague aiding with a countercheck. I believe this process is beneficial because it can help reduce the likelihood of medication errors. (P8, Male, Primary checker, P1 Lines 5-6) |
|  |  | Induce carefulness of checking | We must carefully verify every single detail before administering the medication, especially if we are following the IDC procedures correctly. This is especially important if we strictly adhere to the IDC procedures. (D15, Male, Designated Checker, P5 Lines 160-161) |
|  | Peer‑supported adherence of IDC^#¶^ | Mutual support promotes independence | My colleagues actually do a fantastic job… They follow the IDC process properly, and they do not verbally prompt each other. (D14, Female, Designated Checker, P7 Lines 178-179) |
|  |  | Strictly follow IDC steps | I believe this checking system functions effectively if you adhere to the IDC procedure correctly. (D10, Female, Designated checker, P6 Lines 157) |
|  |  |  | I think a high-quality IDC occurs when two nurses conduct the checks independently, each performing their assessment without verbalising the details of what they are checking. (P7, Male, Primary checker, P5 Lines 118-120) |
|  | Adequate medication knowledge^¶^ | Comprehension of medication knowledge | To minimise medication errors, an IDC requires both parties to have a very clear understanding of the prescription and to be highly familiar with the medication preparation itself. (P1, Female, Primary checker, P4 Lines 112-115) |
| Tools & technology | Digital prescription verification for accuracy improvement^#¶^ | Electronic prescription verification | I believe that the computer system plays an important role in preventing errors during IDC. For instance, the previously mentioned issues with handwritten prescriptions and verbal orders support this assertion. (D5, Male, Designated Checker, P5 Lines 145-146) |
|  |  |  | Since the introduction of IPMOE (Inpatient Medication Order Entry, a computerised medication prescribing, dispensing, and administering system used in Hong Kong hospitals) in our department, I believe it has improved the accuracy of medication delivery during the IDC process by creating a closed loop that ensures the five rights of medication administration. (P5, Male, Primary checker, P3, Lines 90-95) |
|  |  | Scannable code for verification accuracy | I believe that all medications should include a QR code. Scanning it would allow users to verify that they have the correct medication to facilitate the IDC. (D13, Female, Designated checker, P5 Lines 119-121) |
|  |  | Enhancing safety via electronic systems | IPMOE indicates whether the verification was completed by two individuals. If it’s a self-check, only one name appears. Some colleagues want to ensure they are protected, so they will always locate another person to countersign. Simply put, the process does help to improve safety. (P9, Male, Primary checker, P4 Lines 103-108) |
|  |  | Digitised prescriptions improved readability | This is why IDC is important—we examine everything meticulously, word by word… Utilising a computer system is advantageous, as it alleviates some of the challenges associated with implementing IDC. (D12, Female, Designated checker, P6 Lines 162-166) |
|  |  |  | The implementation of the IPMOE system for IDC has certainly made medication checking more convenient. Additionally, the use of preset templates to navigate through the process genuinely reduces the likelihood of errors occurring in the prescription itself. (P4, Female, Primary checker, P5 Lines 139-141) |
|  | Enhancing alertness via electronic systems^#¶^ | Prompted function of electronic systems | Once the information is entered into the CMS (a clinical management system in Hong Kong, developed by the government as an electronic health record platform primarily used by the Hospital Authority (HA) to manage patient information, streamline workflows, and support clinical decision-making), and your name is recorded, you cannot afford to be careless. You are prompted to take the verification process more seriously. (D7, Female, Designated checker, P5 Lines 173-175) |
|  |  |  | I believe that using an electronic system for IDC is quite convenient, as it enables countersigning with either a name or a code. In the event of any issues, there is a clear record within the system; everything is documented and easily traceable. (P6, Female, Primary checker, P4 Lines 103-105) |
|  |  | Automatic IDC alerts in electronic system | Currently, IPMOE does not indicate whether a medication requires IDC. If they provided this information, it might help the IDC process more effectively. (P9, Male, Primary nurse checker, P5 Lines 133-135) |
|  | Adequate facilities streamline workflow^#^ | Dedicated electronic set | Ideally, each nurse should be provided with a dedicated electronic set for medication administration, which would include an iPad, a scanner, and a printer. (D10, Female, Designated checker, P5 Lines 118-122) |
| Task | Clarity of prescription^#¶^ | Clearer orders | I wish for clearer prescriptions; if they were, it would significantly reduce the likelihood of errors. (D3, Female, Designated checker, P2 Line 49) |
|  |  |  | I believe the most crucial factor is ensuring that prescriptions are easily readable and clear, whether entered via IPMOE or recorded in the treatment sheet within the resuscitation room. (P6, Female, Primary checker, P2 Line 52) |
| Process | Independence preventing medication errors#¶ | Independence strengthens accuracy | If we can effectively implement an IDC in a proper and independent manner, it should enhance the accuracy of medication administration and improve overall safety levels. (D4, Male, Designated checker, P1 Lines 21-24) |
|  |  |  | Even among colleagues—irrespective of the trust we place in one another—errors can still arise. This highlights the need for meticulous care and precision in every instance of IDC. (P4, Female, Primary checker, P8 Lines 227-232) |
|  |  | Independent IDC process | With IDC, assuming it truly conducts the process independently, it ought to help reduce errors. (D4, Male, Designated checker, P4 Lines 126-127) |
|  |  |  | Actually, the IDC process is designed to prevent errors that may occur when one person is verifying medication. Having a second individual independently check the work helps minimise the risk of medication errors. (P5, Male, Primary checker, P1 Lines 15-16) |
|  | Efficient pharmacy verification^#¶^ | Timely pharmacy verification | If a pharmacist were always available to verify prescriptions in real time, it would greatly benefit IDC. (D5, Male, Designated Checker, P7 Lines 202-203) |
|  |  |  | We wish to expedite the pharmacy's verification of medications during IDC, particularly for prescriptions originating from the resuscitation room. (P1, Female, Primary checker, P4 Lines 95-101) |
| Outcome | Enhanced patient safety^#¶^ | Safeguard for patient | I believe that IDC is safe, especially from the patient's viewpoint. (D5, Male, Designated Checker, P2 Lines 49) |
|  |  |  | I view IDC as a safeguard for patients. If one nurse makes an error, the other nurse should be able to identify it. (P4, Female, Primary checker, P2 Lines 41-46) |
|  | Protection of healthcare workers^#¶^ | Safety practice of medication  administration | I think that from a coworker's point of view, there is a protective element. Someone with more experience should be the designated checker because they are more likely to know more about medications and be able to notice more details. (D7, Female, Designated checker, P1 Lines 8-10) |
|  |  |  | When administering medications that could be life-threatening if given in excess, particularly on the first occasion, it is natural to feel nervous about making a mistake. Having a colleague double-check with me alleviates my anxiety during the medication administration process and enhances patient safety. (P10, Female, Primary checker, P1 Lines 10-13) |
|  | Total: 15 | Total: 24 |  |

^#:^ Expressed by designated checker; ^¶:^ Expressed by primary nurse.

Supplementary File 2: Barriers, codes, and illustrative quotations of a designated of a designated independent double-checking process for high-alert medication administration using a SEIPS framework.

| Domains of SEIPS framework | Barriers | Codes | Illustrative quotations |
| --- | --- | --- | --- |
| Environment | Distraction^#¶^ | Disturbance | It was mainly due to environmental factors, probably because there were too many distractions. (D8, Female, Designated checker, P6 Line 145) |
|  |  |  | If it’s chaotic and crowded, it might impact the IDC process. Ultimately, I believe the environment plays a crucial role. (P7, Male, Primary nurse, P2 Lines 38-40) |
|  |  | Interruption | Most of the time, there are numerous interruptions during the IDC process, such as the patient’s family, the patient themselves, and our colleagues. (D5, Male, Designated Checker, P6 Lines 174-177) |
|  |  |  | I think that the most significant disruption in the ED stems from patients frequently asking questions during the IDC process. (P8, Male, Primary nurse, P5 Line 129) |
|  |  | Noise | In the ED area, there is already considerable noise due to overhead announcements, beeping machines, and incoming phone calls, all of which can disrupt IDC. (D4, Male, Designated checker, P2 Lines 48-51) |
|  |  |  | When I’m checking a medication, the patient might suddenly shout. In such situations, I might become distracted and glance over for a moment, which subsequently impacts my medication-checking process. (P2, Female, Primary nurse, P4 Lines 97-100) |
|  | Disorganised layout^#^ | Difficulty in locating the patient | Patients in the ED are highly mobile, making it time-consuming to locate them, especially if they have been in X-ray, CT, or other areas. (D7, Female, Designated checker, P8 Line 262-263) |
| Organisation | Inadequate staffing^#¶^ | Insufficient manpower | With shift duty, the issue is that staffing levels on each shift may not always be sufficient. For example, with two APNs serving as in-charge nurses for each shift, there was no third checker available. (D7, Female, Designated checker, P5 Lines 144-145) |
|  |  |  | We need to manage multitasking simultaneously, and the shortage of manpower remains an issue. (P3, Female, Primary nurse, P3 Lines 65-66) |
|  |  | Lack of designated checkers in general duty | I often find that there are not enough designated checkers available during IDC medication administration. (D12, Female, Designated checker, P3 Lines 60-66) |
|  |  |  | During a shift, the team consists mainly of junior staff, or some members may be on their meal breaks, resulting in an insufficient number of designated checkers available. (P10, Female, Primary nurse, P2 Lines 39-41) |
|  |  | Shortage of designated checkers at night shifts | It is often challenging to locate a designated checker to administer IDC medications, particularly during night shifts. (D13, Female, Designated checker, P1 Lines 24-26) |
|  |  |  | At night, it can be very difficult to locate a checker because of insufficient staffing, especially during breaks. (P8, Male, Primary nurse, P4 Lines 113-115) |
|  |  | High turnover | Our staff turnover is exceedingly high. Staff often become familiar with IDC only to leave shortly thereafter, resulting in a constant influx of new colleagues, which complicates matters further. (D9, Female, Designated checker, P7 Lines 185-187) |
|  | Overwhelming workload^#¶^ | Too much workload | When the workload is heavy, it is easy to feel rushed. Even if we remain engaged in the IDC, we may misinterpret information or overlook details. (D1, Male, Designated checker, P3 Lines 78-81) |
|  |  |  | We really have a lot of work going on, and suddenly there are many urgent cases that we need to handle all at once, which can be quite difficult. (P5, Male, Primary nurse, P3 Lines 64-71) |
|  | Unclear guidelines^#¶^ | Lack of clear guides | Sometimes, the guidelines lack clarity regarding which medications require double-checking and which do not. (D9, Female, Designated checker, P6 Lines 165-166) |
|  |  |  | We have materials available to verify whether doctors have made correct prescriptions; however, these materials could benefit from regular review and updates. (P9, Male, Primary checker, P6 Lines 164-166) |
| People | Mental fatigue^#¶^ | Deterioration of metal status | I believe that the demanding nature of shift duties significantly affects IDC, as a lack of alertness may result in missing important details. (D11, Female, Designated checker, P4 Lines 96-98) |
|  |  |  | When working night shifts, my mental state deteriorates; I worry that medication errors are more likely to happen during these hours. (P7, Male, Primary nurse, P4 Lines 92-94) |
|  |  | Reduced attention | Shift work significantly affects my ability to rest adequately. I often feel physically fatigued, and mentally, I struggle with alertness and concentration. This lack of rest ultimately might impact my performance in IDC. (D5, Male, Designated Checker, P5 Lines 132-133) |
|  |  |  | At times, the frequency of the shift cycle is excessive, leading to insufficient rest. Consequently, I find it difficult to concentrate when checking medications. (P11, Male, Primary nurse, P4 Lines 105-106) |
|  | Uncomfortable hierarchy^#^ | Hesitation to speak up | I believe that new colleagues often feel apprehensive about speaking up, particularly when they lack experience. They may hesitate to engage in conversations with doctors or nurses, especially APNs. This can lead to stumbling over their words or feeling uncomfortable, and they might worry that asking additional questions could make them appear foolish. (D7, Female, Designated checker, P3 Lines 88-92) |
|  | Insufficient knowledge^#^ | Unfamiliarity of rotated doctors | Some doctors may be rotating into the department and are not familiar with our protocols or with how medications are usually administered here. As a result, their prescriptions may differ from our usual practice or lead to conflicts. (D9, Female, Designated checker, P2 Lines 55-56) |
|  |  | Inexperienced junior nurses | The junior colleagues may lack the experience or knowledge to perform the IDC medication because they are unfamiliar with the process. (D2, Male, Designated checker, P5 Lines 148-150) |
|  | Perceiving IDC as a burden^#^ | Adding extra work for IDC | We need to find an additional person to double-check the medication, which may introduce an extra step in the process. This does, in fact, create a certain burden. (D5, Male, Designated Checker, P2 Lines 59-60) |
| Tools & technology | Inadequate facilities^#^ | Less-than-ideal environment | The situation becomes quite challenging due to the less-than-ideal environment. The patient has only a bed available, with no table in sight. We find ourselves holding a kidney dish alongside a cup of water. Additionally, we might have some powdered medication. Ultimately, everything is placed on the patient's bed. (D7, Female, Designated checker, P9 Lines 286-289) |
|  |  | Setting restriction | We often find ourselves holding the patient's files while carrying medications in a paper cup as we search for the patient. Frequently, we resort to using the patient's leg as a makeshift surface for placing items. I believe this method of working is far from ideal. (D10, Female, Designated checker, P5 Lines 140-141) |
| Task | Ambiguous information^#¶^ | Confusing prescription | Doctors prescribe “QD” or “daily” for the medication; however, since ED care is available 24 hours a day, I am uncertain whether a QD dose can be administered at any time. The clarity of these prescriptions is lacking. (D11, Female, Designated checker, P4 Lines 98-104) |
|  |  |  | Sometimes, doctors mention only the name of a medication without detailing the dosage, which could affect IDC. (P2, Female, Primary nurse, P3 Lines 82-84) |
|  |  | Unclear handwritten prescriptions | Handwritten prescriptions from doctors can often be challenging to read, as the names of medications, dosages, and routes may not always be clearly legible. (D10, Female, Designated checker, P2 Lines 51-52) |
|  |  |  | For handwritten prescriptions from doctors, elements such as the dosage, route of administration, and preparation of concentration would benefit from clearer writing. (P7, Male, Primary nurse, P2 Lines 51-53) |
|  |  | Inconsistent messages from pharmacy | There was one occasion when the pharmacy confirmed in the electronic prescription that the medication was correct. However, just before administration, they unexpectedly called back to inform us that the medication had actually been prescribed incorrectly. (P1, Female, Primary nurse, P3 Lines 71-72) |
|  | High task variability^#^ | Frequent change of prescriptions | Doctors' decisions can change swiftly. Infusion rates are frequently adjusted, and occasionally a medication is prescribed; however, before administration, it may be altered to an entirely different medication. (D7, Female, Designated checker, P8 Lines 242-244) |
| Process | Time pressure#¶ | Miscommunication during emergencies | In urgent situations where a verbal prescription is issued, the doctor might specify a certain dosage, but a colleague could interpret it as a different dosage. This miscommunication can result in serious misunderstandings. (D5, Male, Designated Checker, P5 Lines 139-141) |
|  |  |  | When a doctor fails to enter the prescription into IPMOE (Inpatient Medication Order Entry, a computerised system for prescribing, dispensing, and administering medication in Hong Kong hospitals) and only provides the name of the medication, the route may not be clearly specified. In such cases, I may be unaware of the correct route, which increases the risk of making a mistake. (P9, Male, Primary nurse checker, P3 Lines 80-81) |
|  |  | Simultaneous demands | What concerns me most is when I am managing one patient and am suddenly called away to verify medication for another patient, which can be perplexing. (D11, Female, Designated checker, P3 Lines 67-68) |
|  |  |  | Usually, while I’m in the middle of IDC, there are family members or patients who come over to ask questions. I have to prepare the meds and answer questions at the same time, which can be disruptive. (P3, Female, Primary nurse, P4 Lines 92-94) |
|  |  | Pressed for time | Sometimes doctors can be inconsiderate, as IDC does take time. Some may complain, asking, “Why does it have to take so long?” (D7, Female, Designated checker, P2 Lines 47-58) |
|  |  |  | Even after I have finished preparing the medications, when I make an overhead announcement or go to find a checker, they may not be able to come immediately. As a result, everything ends up being put on hold while I wait for their arrival. (P2, Female, Primary nurse, P1 Lines 17-19) |
|  |  |  | In the ED, when circumstances become urgent, verbal prescriptions are occasionally issued. However, if we genuinely need to initiate an IDC, by the time we manage to summon the checker, the doctor may have already provided the instruction. This scenario can indeed pose significant risks. (D11, Female, Designated checker, P3 Lines 62-64) |
|  |  |  | Doctors often provide verbal prescriptions rapidly, stating the required medication only once during emergency situations. By the time I locate a designated checker to confirm the information with me, I may not be entirely sure whether the dosage I remember is accurate. (P10, Female, Primary nurse, P2 Lines 52-54) |
|  | Non-compliance of juniors# | Non-adherence on IDC step by juniors | I have noticed that some junior staff occasionally do not adhere fully to the IDC steps and procedures. At times, the sequence is incorrect; for instance, they may neglect to scan the patient’s wristband to verify their identity and, in haste, administer medication directly into the IV access. (D10, Female, Designated checker, P6 Lines 151-154) |
|  |  | Juniors rely on checkers | One concern I have is that junior staff might become overly reliant on the designated checker to the extent that it becomes unclear whether they have engaged in any independent thought during the initial checking step. (D7, Female, Designated checker, P1 Lines 18-19) |
|  | Supervised medication taking# | Assist to take medication | In paediatric cases, we may not be able to administer the medication directly to the child…Instead, we might provide the medication to the parent for them to administer to the child. (D7, Female, Designated checker, P8 Lines 272-276) |
|  | Pharmacy verification problem¶ | Waiting for pharmacy verification | Sometimes, when we call the pharmacy to ask them to verify the medication, it can take ten or even twenty minutes without receiving confirmation. Or they keep telling us that it has already been verified, but it still does not show on our computer system. (P4, Female, Primary nurse, P5 Lines 150-152) |
|  | Total: 16 | Total: 30 |  |

#: Expressed by designated checker; ¶: Expressed by primary nurse.
