# Supplementary material for: Barriers and Facilitators for Medication Safety in Emergency Care Using a SEIPS Model: A Qualitative Study
Source: J Nurs Manag. 2026 Jun 30;2026:3268082. doi: 10.1155/jonm/3268082 (PMC13317145; doi:10.1155/jonm/3268082)
Supplement: Supplementary file 2 — Supporting Information 2 COREQ checklist: Consolidated criteria for reporting qualitative studies (COREQ): 32‐item checklist. [file JONM-2026-3268082-s002.docx]

Consolidated criteria for reporting qualitative studies (COREQ): 32-item checklist.

| **No Item** | **Guide questions/description** | **Reported on page** |
| --- | --- | --- |
| **Domain 1: Research team and reflexivity** | | |
| Personal Characteristics |  |  |
| 1. Interviewer/facilitator | Which author/s conducted the interview or focus group? | 8 |
| 2. Credentials | What were the researcher´s credentials? e.g. PhD, MD | 8 |
| 3. Occupation | What was their occupation at the time of the study? | 8 |
| 4. Gender | Was the researcher male or female? | 8 |
| 5. Experience and training | What experience or training did the researcher have? | 8 |
| Relationship with participants |  |  |
| 6. Relationship established | What did the participants know about the researcher? e.g. personal goals. | 8 |
| 7. Participant knowledge of the interviewer | What did the participants know about the researcher? | 8 |
| 8. Interviewer characteristics | What characteristics were reported about the interviewer/ facilitator? e.g. Bias, assumptions, reasons and interests in the research topic | 8 |
| **Domain 2: Study design** | | |
| Theoretical framework |  |  |
| 9. Methodological orientation and Theory | What methodological orientation was stated to underpin the study? e.g. grounded theory, discourse analysis, ethnography, phenomenology, content analysis | 6 |
| Participant selection |  |  |
| 10. Sampling | How were participants selected? e.g. purposive, convenience, consecutive, snowball | 7 |
| 11. Method of approach | How were participants approached? e.g. face-to-face, telephone, mail, email | 7 |
| 12. Sample size | How many participants were in the study? | 8 |
| 13. Non-participation | How many people refused to participate or dropped out? Reasons? | NA |
| Setting |  |  |
| 14. Setting of data collection | Where was the data collected? e.g. home, clinic, workplace | 7 |
| 15. Presence of nonparticipants | Was anyone else present besides the participants and researchers? | NA |
| 16. Description of sample | What are the important characteristics of the sample? e.g. demographic data, date | 10, Table 2 |
| Data collection |  |  |
| 17. Interview guide | Were questions, prompts, guides provided by the authors? Was it pilot tested? | Table 1 |
| 18. Repeat interviews | Were repeat interviews carried out? If yes, how many? | NA |
| 19. Audio/visual recording | Did the research use audio or visual recording to collect the data? | 8 |
| 20. Field notes | Were field notes made during and/or after the interview or focus group? | 7 |
| 21. Duration | What was the duration of the interviews or focus group? | 8, Table 2 |
| 22. Data saturation | Was data saturation discussed? | 8 |
| 23. Transcripts returned | Were transcripts returned to participants for comment and/or correction? | NA |
| **Domain 3: Analysis and findings** | | |
| Data analysis |  |  |
| 24. Number of data coders | How many data coders coded the data? | 8-9, Supplementary File 1 and File 2 |
| 25. Description of the coding tree | Did authors provide a description of the coding tree? | Supplementary File 1 and File 2 |
| 26. Derivation of themes | Were themes identified in advance or derived from the data? | Supplementary File 1 and File 2 |
| 27. Software | What software, if applicable, was used to manage the data? | 8 |
| 28. Participant checking | Did participants provide feedback on the findings? | NA |
| Reporting |  |  |
| 29. Quotations Presented | Were participant quotations presented to illustrate the themes/findings? Was each quotation identified? e.g. participant number | 11-14, Supplementary File 1 and File 2 |
| 30. Data and findings consistent | Was there consistency between the data presented and the findings? | 14-23 |
| 31. Clarity of major themes | Were major themes clearly presented in the findings? | Figure 2, Supplementary File 1 and File 2 |
| 32. Clarity of minor themes | Is there a description of diverse cases or discussion of minor themes? | Supplementary File 1 and File 2 |

Developed from: Tong, A., Sainsbury, P., & Craig, J. (2007). Consolidated criteria for reporting qualitative research (COREQ): A 32-item checklist for interviews and focus groups. Int J Qual Health Care, 19(6), 349-357. https://doi.org/10.1093/intqhc/mzm042
